# Prevalence of conduct disorder in schoolchildren of Kanke

**DOI:** 10.4103/0019-5545.31579

**Published:** 2006

**Authors:** Sujit Sarkhel, Vinod Kumar Sinha, Manu Arora, Pushpal DeSarkar

**Affiliations:** * Junior Resident, Department of Child and Adolescent Psychiatry, Centre for Cognitive Neurosciences, Central Institute of Psychiatry, Kanke, Ranchi 834006, Jharkhand; ** Associate Professor, Department of Child and Adolescent Psychiatry, Centre for Cognitive Neurosciences, Central Institute of Psychiatry, Kanke, Ranchi 834006, Jharkhand; *** Senior Resident, Department of Child and Adolescent Psychiatry, Centre for Cognitive Neurosciences, Central Institute of Psychiatry, Kanke, Ranchi 834006, Jharkhand; **** Senior Resident, Department of Child and Adolescent Psychiatry, Centre for Cognitive Neurosciences, Central Institute of Psychiatry, Kanke, Ranchi 834006, Jharkhand

**Keywords:** Prevalence, conduct disorder, attention deficit hyperactivity disorder, schoolchildren

## Abstract

**Background:**

Prevalence estimates of conduct disorder, one of the most frequently diagnosed psychiatric conditions in children, vary widely from 0.2% to 8.7%.

**Aim:**

To find out the prevalence of conduct disorder and its DSM-IV subtypes and comorbid attention deficit hyperactivity disorder (ADHD) in 4 schools of Kanke block among students of classes V to X.

**Methods:**

A total of 240 students, selected by stratified random sampling, were subjected to the Schedule for Affective Disorders and Schizophrenia for School Age Children: Present and Lifetime Version (K-SADS-PL) screening interview. Nineteen students who qualified were subjected to conduct disorder and ADHD supplement of K-SADS-PL with additional information from parents.

**Results:**

Conduct disorder was found in 4.58%; the ratio of boys to girls being 4.5:1. Childhood onset was found in 73% and adolescent onset in 27%. Mild conduct disorder was found in 36%, moderate in 64% and severe conduct disorder in none. Comorbid ADHD was found in 36%, hyperactive-impulsive being predominant. Significant difference was found in temperament between students with and without conduct disorder with difficult temperament predominating in the former and easy in the latter (p=0.004). Lying, bullying and cruelty to animals were most frequent symptoms.

**Conclusion:**

The prevalence of conduct disorder was 4.58%, more common in boys, the majority had childhood onset, and one-third had comorbid ADHD.

## INTRODUCTION

The term conduct disorder (CD) refers to a persistent pattern of antisocial behaviour in which the individual repeatedly breaks social rules and carries out aggressive acts that upset other people. DSM-IV mentions CD as one of the most frequently diagnosed conditions in outpatient and inpatient mental health facilities for children.^1^ CD has been separated from the adult diagnosis of antisocial personality in order to acknowledge what psychiatrists believe to be a greater potential for change in the young. CD has been classified along with oppositional defiant disorder and attention-deficit hyperactivity disorder (ADHD) in the attention-deficit and disruptive behaviour disorders section of DSM-IV-TR.^2^

The essential feature of CD is a repetitive and persistent pattern of behaviour in which the basic rights of others or major age-appropriate societal norms or rules are violated. Since its inception in DSM-III, the diagnosis of CD has undergone several modifications. DSM-IV-TR lists 15 criteria grouped into 4 major categories: (i) aggression to people and animals; (ii) destruction of property; (iii) deceitfulness or theft; and (iv) serious violations of rules. Three (or more) of the criteria should have been present for the last 12 months, with at least one criterion present in the past 6 months. The disturbance in behaviour should cause clinically significant impairment in social, academic, or occupational functioning. If the individual is 18 years or older, the criteria for antisocial personality disorder should not be met.^2^

Since the criteria for the diagnosis of CD vary widely, its manifestations at different developmental stages differ and because the databases of different studies are not uniform, the prevalence estimates reported in various studies vary widely.^3^ At one end lies the study of Esser and colleagues^4^ reporting a prevalence of 0.9%, while at the other end is the study by Kashani *et al.*^5^ reporting a prevalence of 8.7%. DSM-IV reports a prevalence in males of 6%–10% and in females of 2%–9%.^1^ The ratio of males to females with CD is lower for the adolescent-onset type than for the childhood-onset type.^2^ Among Indian studies, Deivasigamani^6^ has reported the prevalence of CD to be 11.13%, Sarkar *et al.*^7^ reported the prevalence rate of antisocial behaviour to be 7.1% while recently Srinath *et al.*^8^ have reported a prevalence as low as 0.2%. ADHD is a common comorbidity in children with conduct disorder. In addition, studies suggest that CD is more severe and persistent when children also exhibit ADHD.^9^ Satterfield and Schell^10^ found that, in hyperactive boys, only one conduct problem was necessary to predict serious antisocial behaviour in adolescence and childhood. Hence, comorbid ADHD was also looked for in students with conduct disorder. Leaving out one or more key informants was one of the major drawbacks of several other studies,^5^–^6^,^11^ since individuals with CD are likely to minimize their conduct problems and the clinician often must rely on additional informants.^2^ The current study aimed to overcome this drawback by collecting information from parents, teachers and the student himself.

Conduct disorder poses a major problem at the personal and social level.^12^ Keeping in view the serious nature of the problem and the dearth of comprehensive studies in this regard, the current study was planned. Schools, being a major influence on the growing and developing mind of the child, provide an ideal setting to carry out such a study. The aim of the current study was to find out the prevalence of CD, its DSM-IV subtypes and comorbid ADHD among schoolchildren of Kanke. Moreover, temperament, an important predictor of subsequent development of CD which was left out in the majority of previous studies, was to be assessed which could play a pivotal role in planning early intervention.

## METHODS

The present study was conducted in various schools of Kanke, the block headquarters of Kanke Block of Ranchi District. Out of 15 schools of Kanke, 4 schools were chosen by means of simple random sampling for the present study. Data collection was done in two stages. In the first stage, screening was done and in the second stage, a detailed diagnostic interview was conducted.

Sampling was done from a total pool of 1690 students. The entire universe was stratified based on Standard, from V to X roughly corresponding to 10–15 years of age. Ten students from each standard were selected by random sampling for screening. Hence, a total of 60 students from each school were selected constituting a grand total of 240 students which included 132 boys and 108 girls. Students with mental retardation and psychosis were excluded from the study. Each student was interviewed personally applying the CD screening section of Schedule for Affective Disorders and Schizophrenia for School-Age Children-Present and Lifetime Version (K-SADS-PL).^12^ The responses were corroborated separately from the respective class teachers. Students who crossed the cut-off score of 3 in at least one out of five screening items (lying, truancy, physical fights, bullying and non aggressive stealing) in the interview were selected for detailed assessment in the second stage.

In the second stage, the CD and ADHD supplement of K-SADS-PL^13^ were applied along with the sociodemographic and clinical data sheet. Additionally, temperament of the child was assessed across 3 different constellations of temperament as defined by Stella Chess and Alexander Thomas.^14^ There were 9 different temperamental categories, namely, activity level, rhythmicity, approach-withdrawal, adaptability, threshold of responsiveness, quality of mood, distractibility, attention span, and persistence. Three different temperamental constellations, i.e. easy, slow-to-warm-up and difficult were based on various combinations of these categories which were assessed qualitatively with the help of the checklist designed by Chess and Thomas.^14^ For this purpose, home visit was made and the student and the parents were interviewed separately. Finally, based on overall impression, a DSM-IV diagnosis of CD (with various subtypes) and comorbid ADHD was made.

### Statistical analysis

Statistical analysis was done using the Statistical Package for Social Sciences (SPSS, Inc., Chicago, Illinois) version 11.0. Descriptive statistics was used to describe the sample in terms of sociodemographic and clinical characteristics. Non-parametric tests (χ^2^ and Mann-Whitney U) were used to compare between groups. In this study, a level of significance (α) of < 0.05 (2-tailed) was taken to consider a result (group difference) statistically significant.

## RESULTS

Out of 240 students, 19 were identified by K-SADS-PL screening interview. Data was collected from these students. Eleven students were finally diagnosed of conduct disorder out of which 9 were boys and 2 were girls. The prevalence of CD (Table 1) in the current study was found to be 4.58%, prevalence among boys being 6.81% (n=9) and girls being 1.85% (n=2). The ratio between boys and girls with conduct disorder was found to be 4.5:1. In childhood onset type, the ratio of boys to girls was found to be 7:1 while in adolescent onset type, it was reduced to 2:1.

**Table 1:** Prevalence of conduct disorder in schoolchildren of Kanke

Diagnosis	*n*	Prevalence (%)
Conduct disorder	11	4.58
Sex		
Boys	9	6.81
Girls	2	1.85
Onset		
Childhood	8	72.72
Adolescent	3	27.27
Severity		
Moderate	7	63.63
Mild	4	36.36
Severe	0	00.00
Conduct disorder with comorbid ADHD	4	36.36
Hyperactive impulsive type	3	75.00
Combined type	1	25.00
Inattentive type	0	0.0

ADHD attention-deficit hyperactivity disorder

Childhood onset of CD is characterized by onset of at least one criterion before 10 years of age and if none is present by this age, it qualifies for adolescent onset.^2^ In the present study, childhood onset (72.72%) of CD was found to be more common in comparison to adolescent onset (27.27%). CD of moderate severity was found in 63.63%, mild severity in 36.36%, while none had severe CD. Among students with conduct disorder, 36.36% (n=4) had comorbid ADHD diagnosis, 75% (n=3) of whom were of predominantly hyperactive-impulsive type and 25% (n=1) were of combined type. None was found to have predominantly inattentive type of ADHD.

Comparison of sociodemographic variables between students with and without CD revealed that both the groups were comparable in every aspect except temperament. A significant difference in temperament between the groups was found (p=0.004). Difficult temperament was found in 72.7% (n=8) of the students with conduct disorder while none had difficult temperament in the other group. Likewise, easy temperament was found in 37.5% (n=3) of those who did not have conduct disorder but was absent in children with CD (Table 2).

**Table 2:** Comparison of sociodemographic and clinical variables between students with and without conduct disorder

Variables	Conduct disorder present (*n*=11) (M ± SD) *n* (%)	Conduct disorder absent (*n*=8) (M ± SD) *n* (%)	Analysis		
			
			χ^2^/Mann–Whitney U	p
Age (years)	14.72 ± 1.10	13.87 ± 0.83	1.712	0.087
Sex				
Male	9 (81.8)	4 (50.0)		
Female	2 (18.2)	4 (50.0)	2.170	0.141
Years of schooling	8.63 ± 1.80	8.25 ± 1.28	0.597	0.550
Subjects failed last year	4.18 ± 1.94	2.62 ± 1.18	1.687	0.092
Number of siblings	4.54 ± 1.43	4.12 ± 0.83	0.851	0.395
Substance use in students				
Nil	5 (45.5)	7 (87.5)		
Nicotine dependence	4 (36.4)	1 (12.5)		
Nicotine dependence and alcohol abuse	2 (18.2)	0 (0.0)	3.753	0.153
Income of parents				
Up to Rs 5000 p.m	5 (45.5)	6 (75.0)		
Above Rs 5000 p.m.	6 (54.5)	2 (25.0)	1.659	0.198
Substance use in parents				
Nil	5 (45.5)	7 (87.5)		
Cannabis dependence	1 (9.1)	0 (0.0)		
Alcohol abuse	4 (36.4)	1 (12.5)		
Alcohol dependence	1 (9.1)	0 (0.0)	3.753	0.289
Temperament				
Difficult	8 (72.7)	0 (0.0)		
Slow-to-warm up	3 (27.3)	5 (62.5)		
Easy	0 (0.0)	3 (37.5)	11.308	0.004*

* Significant group difference between easy and difficult temperament at p<0.05 (2-tailed)

In the present study, lying, bullying and cruelty to animals were most commonly found symptoms whereas, aggressive stealing, forced sexual activity, fire setting and running away overnight were absent in students with CD (Table 3).

**Table 3:** Distribution of symptoms of conduct disorder among students who were diagnosed of the disorder

Symptoms	Nil (%)	Sub-threshold (%)	Threshold (%)
Bullying	0 (0)	1 (9.1)	10 (90.9)
Cruelty to animals	0 (0)	1 (9.1)	10 (90.9)
Physical fights	0 (0)	3 (27.3)	8 (72.7)
Physical cruelty to persons	0 (0)	3 (27.3)	8 (72.7)
Use of weapon	4 (36.4)	4 (36.4)	3 (27.3)
Aggressive stealing	11 (100)	0 (0)	0 (0)
Forced sexual activity	11 (100)	0 (0)	0 (0)
Vandalism	0 (0)	8 (72.7)	3 (27.3)
Fire setting	11 (100)	0 (0)	0 (0)
Lying	0 (0)	0 (0)	11 (100)
Non-aggressive stealing	0 (0)	2 (18.2)	9 (81.8)
Breaking and entering	10 (90.9)	1 (9.1)	0 (0)
Truancy	0 (0)	3 (27.3)	8 (72.7)
Staying out at night	2 (18.2)	1 (9.1)	8 (72.7)
Running away overnight	11 (100)	0 (0)	0 (0)

Symptoms have been described as nil, sub-threshold and threshold as per K-SADS-PL according to increasing severity

When various symptom profiles were compared between students with and without CD, no significant difference was found in lying and truancy between the two groups (Table 4).

**Table 4:** Group difference of individual items between students with and without conduct disorder

	Conduct disorder			
			
Items	Present (*n*=11) (M ± SD)	Absent (*n*=8) (M ± SD)	Mann-Whitney U (Z) (df=1)	p
Physical fights	2.72 ± 0.46	2.00 ± 0.00	3.086	0.002**
Bullying	2.90 ± 0.30	1.62 ± 0.51	3.782	0.000**
Use of weapon	1.90 ± 0.83	1.00 ± 0.00	2.693	0.007**
Physical cruelty to persons	2.72 ± 0.46	1.87 ± 0.35	3.129	0.002**
Cruelty to animals	2.90 ± 0.30	1.75 ± 0.46	3.773	0.000**
Vandalism	2.27 ± 0.46	1.12 ± 0.35	3.599	0.000**
Lying	3.00 ± 0.00	2.62 ± 0.74	1.704	0.088
Non-aggressive stealing	2.81 ± 0.40	2.12 ± 0.83	2.077	0.038*
Truancy	2.72 ± 0.46	2.62 ± 0.51	0.461	0.645
Staying out at night	2.54 ± 0.82	1.25 ± 0.46	0.003	0.005**

* Significant at p<0.05 (2-tailed)

** Significant at p<0.01 (2-tailed)

## DISCUSSION

### Methodological issues

Besides providing information that can be compared with the child′s behaviour at home, school can be used as an alternative setting for carrying out interventions and allows sampling of a broader spectrum and area of behaviour.^15^ Moreover, externalizing problems get reported more often by teachers and internalizing problems more often by the parents.^16^ Keeping these views in mind, school was chosen as a platform to obtain samples for study. This has been done in several other studies.^6^,^17^ The combined sample from four schools consisting of 240 children that were screened comprised of an almost equal proportion of girls and boys. This ensured proportional representation of both sexes. Children are essential informants regarding CD because their covert acts are not easily noticed by adults.^18^ Because individuals with CD are likely to minimize their conduct problems, the clinician often must rely on additional informants.^2^ In the present study, information from child, teacher and both parents was used and the interviewer′s best judgement was applied to arrive at a final conclusion. This was an improvement over several other studies which left out one or more key informants.^5^,^6^,^11^ To encompass a broad array of age-typical manifestations, an age range of 10–15 years was targeted. This was done in some studies^19^,^20^ but not in others.^5^,^6^ The K-SADS-PL^13^ used in this study gives the interviewer sufficient liberty in adapting questions to suit the respondent, and allows him to make further probes depending on the response. It adheres closely to the diagnostic criteria laid down in DSM-IV and generates diagnosis accordingly.

### Comparison with other studies

The prevalence of CD in the current study was found to be 4.58%, prevalence among boys being 3.75% (n=9) and girls being 1.85% (n=2). This was in agreement with several other studies.^21^,^25^ Studies by Kashani *et al.,*^5^*Cohen et al.*^11^ and Deivasigamani^6^ have found higher rates while that by Esser *et al.*^4^ revealed lower rate of prevalence. This difference could be attributed to variations in age group, informants, diagnostic system, diagnostic tools and sampling techniques. The ratio between boys and girls with CD was found to be 4.5:1 which was similar to findings of Rutter *et al.,*^26^ Offord *et al.*^23^ and Feehan *et al.*^27^ In childhood onset type, the ratio of boys to girls was found to be 7:1 while in adolescent onset type, it was reduced to 2:1. This finding was in agreement with DSM-IV-TR.^2^ Several other studies have also supported the narrowing of sex differences during adolescence.^11^,^23^ In the current study, childhood onset (72.72%) of CD was found to be more common in comparison to adolescent onset (27.27%). This was in agreement with several Indian studies that found trends of increasing prevalence in 5–10-year-olds.^19^,^28^ Predominance of difficult temperament among students with conduct disorder could also explain childhood onset in the majority. Other sources^12^ mention adolescent onset type as more common.

In terms of severity, CD of moderate severity was found in 63.63%, while that of mild severity was found in 36.36%. A key finding has been the absence of severe CD in the study sample. Severity of CD is a predictor of poor outcome, and academic underachievement and school dropout is one of the common outcomes of CD.^12^ The overlap between externalizing difficulties and academic failure clearly is sizeable and important.^29^ This could explain the above finding.

In the present study, among the students with conduct disorder, 36.36% had comorbid ADHD. This was similar to the figures reported by Biederman *et al.*^30^ and Cohen *et al.*^11^ Amongst those with comorbid ADHD, 75% were of hyperactive-impulsive type and 25% were of combined type. This was in agreement with DSM-IV-TR.^2^ The current study found a significant difference in temperament between the group with and without CD with difficult temperament predominating in the former. Difficult temperament was found in 72.7% (n=8) of students with conduct disorder while none had difficult temperament in the other group which is in agreement with the finding of Chess and Thomas^14^ and Caspi *et al.*^31^ Components of difficult temperament as predictors of CD have also been found in studies by Sanson and Prior^32^ and Graham *et al.*^33^ Temperament, which emerged as a major predictor of CD, was not assessed in most studies.

In the present study, symptoms such as aggressive stealing, forced sexual activity, fire setting and running away overnight were absent in students with CD whereas breaking and entering was present only in one student. This was similar to the findings of Rao *et al.*^34^ who concluded that fire setting, trouble with the law and sexual promiscuity were not found in India. Moreover, absence of severe CD in the sample could explain the above finding. Lying, bullying and cruelty to animals were most commonly found symptoms, again in keeping with the findings of Rao *et al.*^34^ When distribution of various symptoms was compared between students with and without CD, no significant difference was found in lying and truancy between the two groups. This reflects that lying and truancy could be the common presentation of several causes, CD being one of them. These two features are also common causes of school problems and reflect the teachers' inherent bias against those exhibiting these features. Consequently, screening questionnaires that include these two items would invariably result in false-positivity to some extent.

A trend was reflected in the number of subjects failed last year when comparison was made between the groups with and without CD with higher failures in the former. Thus, academic difficulty was higher in the group with CD. This was in agreement with several studies demonstrating academic underachievement in CD.^29^,^35^

The present study had certain limitations. Inherent disadvantages of sampling in schools remained. Subjects with severe CD, being probable school dropouts, were automatically left out. Certain sociodemographic and clinical variables such as peer adjustment, environment in neighbourhood, parenting style and intelligence, which were not probed, kept room for future improvement.
